# Patient-Reported Outcome Measures in Routine Pediatric Clinical Care: A Systematic Review

**DOI:** 10.3389/fped.2020.00364

**Published:** 2020-07-28

**Authors:** Sumedh Bele, Ashton Chugh, Bijan Mohamed, Lorynn Teela, Lotte Haverman, Maria J. Santana

**Affiliations:** ^1^Department of Community Health Sciences, Cumming School of Medicine, University of Calgary, Calgary, AB, Canada; ^2^Postgraduate Medical Education, Harvard Medical School, Harvard University, Boston, MA, United States; ^3^Department of Pediatrics, Cumming School of Medicine, University of Calgary, Calgary, AB, Canada; ^4^School of Physical Therapy, Western University, London, ON, Canada; ^5^Psychosocial Department, Emma Children's Hospital Amsterdam UMC, University of Amsterdam, Amsterdam, Netherlands

**Keywords:** patient-reported outcome measures (PROMs), patient-centered care, pediatrics, routine clinical care, health-related quality of life (HRQOL)

## Abstract

**Introduction:** Integration of patient-reported outcome measures (PROMs) in routine clinical care is growing but lacks consolidated evidence around its impact on pediatric care. This systematic review aims to evaluate the impact of integrating PROMs in routine pediatric clinical care on various outcomes in pediatric clinical care.

**Data Sources:** MEDLINE, Embase, CINAHL, PsycINFO, and Cochrane Library. Web of Science database was searched selectively to ensure extended coverage.

**Study Selection:** We included longitudinal studies reporting on the integration of PROMs in routine pediatric clinical care of chronic diseases. Studies in languages other than English, published prior to the year 2000, and reporting on secondary data were excluded.

**Data Extraction:** Two reviewers independently extracted data from included studies. Extracted data included citation of each study, type of healthcare setting, location of the study, characteristics of patient population, type of chronic disease, name and type of PROM, mode of administration, and reported outcomes.

**Results:** Out of 6,869 articles, titles and abstracts of 5,416 articles and full text of 23 articles were screened in duplicate. Seven articles reporting results from six studies met eligibility criteria. Integration of PROMs increased the identification and discussion around health-related quality of life (HRQOL), especially in psychosocial and emotional domains, but showed mixed results with the impact on quality of care. No studies assessed the impact of integrating PROMs on healthcare utilization.

**Limitations:** Due to significant heterogeneity in the studies, a meta-analysis was not conducted.

**Conclusions:** Integrating PROMs could have a positive impact on HRQOL; however, further studies are required to determine the impact of PROMs in routine pediatric clinical care.

## Introduction

Improvements in treatment options and reduction in infectious diseases in the last century has dramatically enhanced overall survival among children, but this has increased the prevalence of chronic diseases among children and youth (1). Depending on the definition used, almost 13–27% of children are suffering from chronic diseases, which can influence their health and academic outcomes, such as absenteeism, concentration, and grades (2). Chronic diseases have considerable financial and organizational consequences for healthcare systems as well (3, 4).

Healthcare systems around the world are also shifting toward a patient-centered care (PCC) model, which incorporates and responds to individual patient and family preferences, needs, and values (5). Patient–clinician interaction is essential to optimize chronic disease management; therefore, integration of patient-reported outcome measures (PROMs) in routine clinical care is touted as an effective way to steer healthcare toward a PCC model and provide integrated care (6, 7). PROMs are self-completed questionnaires that have been standardized and validated and assess the following health domains: physical, emotional, social, functional, overall well-being, and health-related quality of life (HRQOL) (8, 9). HRQOL can be defined as “how well a person functions in their life and his or her perceived well-being in physical, mental, and social domains of health” (10).

PROMs capture the impact of a disease and/or treatment on the patient, and therefore, their use in routine clinical care is on the rise (11). According to the framework of Santana and Feeny (12) integration of PROMs in routine clinical practice enhances communication among healthcare providers, patients, and relatives, leading to better chronic care management and patient outcomes. This is especially pertinent as the rising prevalence of chronic diseases is leading to higher utilization of health services, adding further stress on our healthcare systems (13–15). Without a universal definition of chronic disease in pediatrics, there is a large degree of variation in the use of the term chronic disease (16). In this study, a comprehensive definition of chronic conditions developed through a national consensus in the Netherlands is used, which states that “a chronic condition in childhood”: (1) occurs in children aged 0 up to 18 years; (2) its diagnosis is based on medical scientific knowledge and can be established using valid methods according to the professionals; (3) it is not (yet) curable and; (4) it has been present for longer than 3 months or it has occurred three times or more during the past year and will probably recur again” (17).

Evidence from the adult population suggests that PROMs help to identify physical and psychosocial issues in patients, which facilitates discussion of those issues during medical visits or inpatient stay (11, 12). As a result, addressing physical and psychosocial issues improves clinical outcomes and, consequently, HRQOL. In addition, integration of PROMs in routine clinical care also impacts referral rates, consultation times, and clinical outcomes and is considered a strategy to improve overall quality of care (18).

Age-related variations in the comprehension of health concepts and differences in age-related vocabulary complicate formatting and design of PROMs for the pediatric population and have led to the creation of multiple forms of pediatric PROMs (19, 20). Despite these challenges, there is evidence to suggest that children above the age of 8 are able to reliably report their health status if PROM instruments are developed by accommodating specific content related to their vocabulary and cognitive and reading abilities (21, 22). Although the impact of PROMs in adults is well-established, research in pediatric settings has lagged. This is concerning as frequent hospitalizations, medical visits, and subsequent school absenteeism negatively impact the psychosocial development of children and youth, resulting in poor clinical outcomes and lower HRQOL (13–15).

PCC in pediatric clinical settings include family caregivers since questions from the medical team are usually directed to the patient's family/caregiver, who acts as the primary agent in the delivery of the patient's care. Proxy-PROMs completed by the caregivers are not actually “patient-reported” because caregivers or healthcare providers of pediatric patients are asked to report on a child's experiences of subjective constructs, such as emotional state, level of satisfaction, or pain severity (23). The evidence of agreement between pediatric patient's self-reported and proxy-reported outcomes is tenuous (23–26). However, considering the family caregiver's role in pediatric care, proxy-PROMs still provide crucial information about a child's health and functioning to healthcare providers, which assist them in providing appropriate clinical care (22, 23).

The objective of this systematic review is to consolidate the evidence to evaluate the impact of using PROMs as an intervention in routine clinical care for pediatric patients with chronic diseases. The outcomes of interest for this systematic review include healthcare utilization, HRQOL, clinical outcomes, and quality of care. Since chronic diseases are associated with adverse long-term outcomes, they require complex care (27). Pediatric patients with chronic diseases receive long-term care usually with the same providers or at the same facilities which creates an opportunity to evaluate the impact of integrating PROMs on routine clinical care over an extended time period or for multiple clinical encounters. Therefore, this systematic review will only focus on the chronic diseases. We also wanted to synthesize evidence using a patient-oriented research approach; therefore, we have engaged patient-advisers in this review.

## Methods and Analysis

### Design

The protocol of this systematic review has been registered with PROSPERO (Registration number: CRD42018109035) and has been published in a peer-reviewed journal (28). The preferred reporting items for systematic review and meta-analysis (PRISMA) guidelines (29) led the administration and reporting of this review.

### Search Strategy

Based on the research question, MeSH (Medical Subject Headings) terms, their variations, and other keywords were used to capture studies focusing on three concept clusters: population, intervention, and outcomes. First, with the support from a medical sciences librarian, the search strategy for MEDLINE was developed. Range of keywords was used to build a robust search strategy focusing on each domain. To capture studies focusing on pediatric populations (18 years or younger), keywords such as “child,” “adolescent,” “Child Health Services,” and “Child, hospitalized” were used. To identify studies implementing PROMs in clinical care, keywords such as “patient-reported outcomes,” “patient outcome assessment,” and a combination of “outcome” and “measures” along with the associated abbreviations (PRO, PROM) were used. Considering the variety of medical outcomes of interest for this systematic review, keywords such as “visits to emergency services,” “length of stay,” “patient admission,” “patient readmission,” “nurse–patient relations,” and “physician–patient communication” were used to capture studies focusing on the overall utilization of healthcare services. Furthermore, keywords associated with patient outcomes such as “HRQOL” and “quality of life,” along with keywords such as “Quality of Health Care” and “Quality Indicators, Health Care” associated with the quality of healthcare were used. To develop a comprehensive search strategy, terms within each concept cluster were combined using Boolean operator “OR,” then all three concept clusters were combined using the Boolean operator “AND.” To further refine this search strategy, it was applied in MEDLINE (Ovid interface, 1950 onward) database to randomly select 100 abstracts. These 100 abstracts were reviewed to assess the specificity of the search strategy. Lack of some keywords in this initial search strategy resulted in inclusion of studies focusing on validation of PROMs or use of PROMs in drug label claims, which do not adhere to the aim of this systematic review. Therefore, screening this random sample of abstracts helped us to refine the search strategy and set the inclusion and exclusion criteria. Final search strategy is provided in Appendix 1.

The final search strategy was applied to search MEDLINE (Ovid interface, 1950 onward) database and adapted to the syntax of other databases including Embase (Ovid Interface, 1974 onward), CINAHL Plus with Full Text (EBSCOhost interface, 1982 onward), PsycINFO (Ovid interface, 1803 onward), and Cochrane Library (Ovid Interface, 1991 onward). To ensure more complete coverage of the literature, the reference list of included studies was hand-searched. Furthermore, included studies were searched in Web of Science (Thomson Reuters) database to find additional studies citing these studies. Integration of PROMs in routine clinical care initiated after the year 2000, so a time limit was applied to only include the studies post-year 2000. To avoid missing any studies due to their design, study design limits were not imposed on the search; however, at the screening stage, studies without any control/comparison group were excluded. Due to the limited capacity in translating non-English articles, English language filter was applied.

### Study Screening and Selection

To reduce the possibility of excluding relevant articles and to mitigate individual bias, three of the team members (SB, BM, and AC) worked in pairs to independently screen titles and abstracts of all studies against our prespecified inclusion and exclusion criteria. Disagreements were resolved through discussion with a third reviewer. Senior authors (MS and LH) were consulted if disagreement persisted.

Studies were included if they primarily focused on the implementation and use of PROMs in pediatrics and only if PROM questionnaires were completed by pediatric patients with chronic diseases and were based on primary data and reported at least one of the outcomes of interest (HRQOL, symptom control, mortality, healthcare utilization, quality of care). Studies were excluded if they reported the use of PROMs for acute diseases, dental problems, pharmaceutical testing, or surgical outcomes assessment or if they reported secondary data including descriptive studies and reviews. We excluded studies where only proxy-PROMs were used for children above the age of 8. Studies using only proxy-reported PROMs were included if they were used for children below the age of 8. Studies in languages other than English or those published prior to year 2000 were also excluded.

Throughout the review, EndNote Reference Management Software (V.8) was used to manage literature search results including removal of duplicate references and screening of all the references.

### Data Extraction

Two reviewers (SB, AC) used a standardized data extraction form to independently extract data from studies included in the final analysis. The extracted data included citation of each included study, type of pediatric healthcare setting, location of the study, characteristics of patient population, type of chronic disease, name and type of PROM, mode of administration, and reported outcomes (Table 1).

**Table 1:** Overview of the characteristics of included studies.

**References**	**Country of origin**	**Study objective**	**Design**	**Duration**	**Chronic disease**	**Participants**		**PROM and type**	**PROM frequency and mode of administration**	**Evaluated outcomes**	**Quality score**
						**Patients/family members**	**Health care providers**				
(30)	Netherlands	To test the effects of monitoring and discussing HRQOL in adolescents with type-1 diabetes in a multicenter randomized controlled trial	Randomized controlled trial	12 Months	Type-1 diabetes	Pediatric patients (81)	Pediatricians (7)	Generic: PedsQL; Disease specific: PedsQL Diabetes—specific module	Three consultations (Prior to regular visits) Electronic	Physical and psychosocial well-being Depression Diabetes-specific family conflict Satisfaction with care Glycemic control	22
(31)	Netherlands	To investigate the effectiveness of an intervention that provides HRQOL scores of the patient (PRO tool) to the oncologists	Sequential cohort study	45 Months	Cancers (Including leukemia, lymphoma, brain tumor, solid tumor, bone tumor etc.)	Pediatric patients (193)	Pediatric oncologists (N/R)	Generic: PedsQL (for 8–18); PedsQL parent proxy (For 6–7); TAPQOL (For 0–5)	Three consultations (Prior to consultation) Electronic	HRQOL Communication about HRQOL Domains Identification of HRQOL problems Consultation duration Referral rate	16
(32)	Netherlands	To investigate the content including type and number of psychosocial topics discussed during a pediatric oncology consultation	Sequential cohort study	45 Months	Cancers (Including leukemia, lymphoma, brain tumor, solid tumor, bone tumor etc.)	Pediatric patients (193)	Pediatric oncologists (N/R)	Generic: PedsQL (for 8–18); PedsQL parent proxy (For 6–7); TAPQOL (For 0–5)	Three consultations (Prior to consultation) Electronic	Discussion on HRQOL domains	16
(33)	Netherlands	To investigate the effectiveness of a web-based intervention that provided an ePRO to the pediatric rheumatologists during consultation	Sequential cohort study	13 months (3 months: control, 10 months: intervention)	Juvenile idiopathic arthritis	Pediatric patients (176)	Pediatric rheumatologists (5)	Generic: PedsQL (for 8–18); PedsQL parent proxy (For 6–7); TAPQOL (For 0–5); Disease specific: Self-composed questionnaire based on DISABKIDS arthritis module; CHAQ—Dutch version	Before each consultation (1,2) Electronic	Communication about HRQOL Satisfaction among parents Satisfaction among health care providers	15
(34)	USA	To determine whether feeding back patient-reported outcomes (PROs) to providers and families of children with advanced cancer improves symptom distress and health-related quality of life.	Randomized controlled trial	~5 months (20 weeks)	Cancers (Patients with at least 2-week history of progressive, recurrent or non-responsive cancer or for whom there was a decision not to pursue cancer-directed therapy)	Pediatric patients (98)	69 Oncologists (61: Physicians, 8: nurses)	Generic: an adapted version of the validated MSAS; PedsQL 4.0; An overall sickness questionnaire; Disease specific: PQ-MSAS	Ranged from once a week to once a month Electronic	HRQOL Satisfaction with the PROMs integration program Identification of HRQOL related issues Referral rate Consultation duration	20
(35)	Germany	To assess both the feasibility and acceptability of electronic PROs	Cohort study (+ Mixed methods)	12 months	Asthma, Diabetes or rheumatic arthritis	Pediatric patients (312)	N/A, but eight subspecialist pediatricians were selected for qualitative study	Generic: Item bank approach but Kids-CAT tool followed the domain structure of KIDSCREEN-27 questionnaire	Four times (baseline, 3,6 and 12 months) Electronic	Communication about HRQoL Domains Consultation duration Impact on patient-physician communication Satisfaction with the PROMs integration program	12
(36)	Spain	To assess whether the systematic monitoring of HRQOL via internet in clinical practice in Spanish pediatric patients with T1DM helps improve their daily life with diabetes.	Randomized control trial	12 months	Type-1 diabetes	Pediatric patients (119)	Endocrinologists (7)	Generic: KIDSCREEN-27 and KIDSCREEN-10; Disease specific: SDQ	Four times (baseline, 3, 6, and 12 months) in intervention group and 1 and 12 months in control group Electronic	HRQOL	21

*PRO, Patient-reported Outcome; PROM, Patient-reported outcome measure; HRQOL, Health-related quality of life; PedsQL, Pediatric Quality of Life; TAPQOL, TNO-AZL Preschool children Quality of Life; CHAQ, Childhood Health Assessment Questionnaire; PQ-MSAS, PQ Memorial Symptom Assessment Scale; SDQ, Strengths and Difficulties Questionnaire*.

### Methodological Quality of Studies

Originally, we had anticipated identifying unvalidated PROMs, so we had planned to assess the risk of bias and methodological quality of included studies using the COnsensus-based Standards for the selection of health status Measurement INstruments (COSMIN) checklist (37). However, all studies included in this review used validated PROM measures; therefore, we decided to assess the methodological quality using the Downs and Black checklist (38). The Downs and Black checklist consists of 27 items distributed between five subscales: (1) reporting; (2) external validity; (3) internal validity-bias; (4) internal validity-confounding; and (5) power (38). Each item on the checklist is scored as either 0 or 1, except the item on principal confounders, which can be scored as either 0, 1, or 2. The total score on this checklist was originally 32 points, but several studies have shown difficulties in interpreting the last item on power calculation, so it was recommended to dichotomize that item to give it a score of either 1 if sufficient power calculations were reported or 0 if otherwise (39). We also followed this approach. Therefore, the adjusted total maximum score of the checklist in this review was 28. Risk of bias in individual studies was assessed independently by two reviewers (SB, LT), and discrepancies were resolved by discussion to reach a consensus.

### Data Synthesis

**Figure 1:**
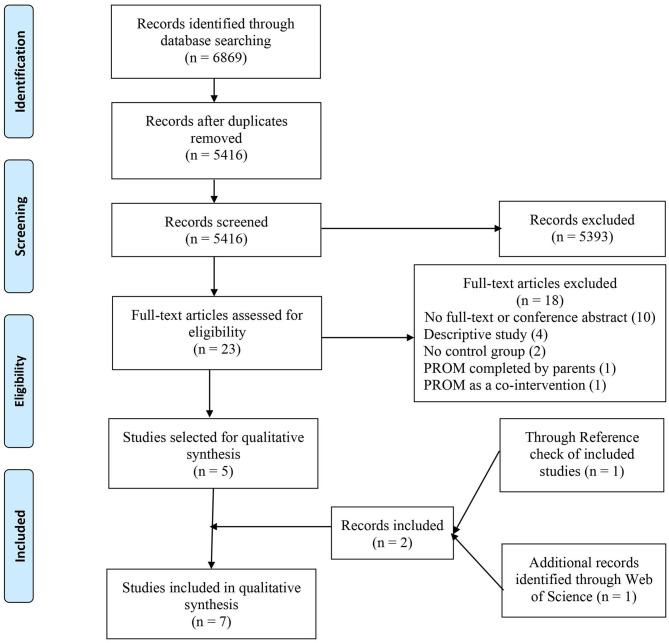
The preferred reporting items for systematic review and meta-analysis (PRISMA) flow diagram of identification and selection process of studies.

A PRISMA flow diagram was generated (Figure 1) to report the number of studies identified, screened, and included in the final synthesis. Extracted data were summarized and tabulated (Table 2). Quantitative data were not reported by most of the included studies, and there was considerable heterogeneity in terms of the study designs, study population, type of chronic disease, and reported outcomes, making it impossible to perform a meta-analysis. A descriptive summary of included studies and a narrative analysis of the results are presented. Finally, we presented extracted data and findings of our systematic review to our patient-advisers.

**Table 2:** Summary of the impact of integrating patient-reported outcome measures on routine pediatric clinical care in intervention group compared to control group.

**References**	**Country of origin**	**HRQOL**	**Healthcare utilization**	**Quality of care**	**Patient Outcomes**	**Communication about HRQOL Domains**	**Average consultation duration**	**Identification of HRQOL problems**	**Satisfaction (with intervention)**	**Referrals**
(30)	Netherlands	=	NR	NR	=	↑^*^	NR	NR	↑*	NR
(31)	Netherlands	↑^*^	NR	NR	NR	↑*	↓	↑	↑	=
(32)	Netherlands	NR	NR	NR	NR	↑	↓	NR	NR	NR
(33)	Netherlands	NR	NR	NR	NR	↑	NR	NR	= (↑ Among providers)	↑
(34)	USA	↑	NR	NR	NR	NR	↓↑	↑	↑	↑
(35)	Germany	NR	NR	NR	NR	↑	↓↑	↑	↑	NR
(36)	Spain	↑*	NR	NR	=	NR	NR	NR	NR	NR

*HRQOL, Health-related quality of life*.

↑, Increase; ↓, Decrease; ↓↑, Mixed results;

* *Statistically significant; NR, Not reported; =, No change*.

### Soliciting Patient-Advisers' Perspectives

To assess the relevance of the results of this systematic review, the patient-advisers were consulted. These patient-advisers included three children/youth and their mothers. Youngest child adviser was 8 years old, and the oldest youth adviser was 18 years old. These three children/youth advisers have the experience of living with chronic conditions, and their mothers have experience of caring for them. Considering their lived experience, we engaged them in our systematic review. Our approach was to present them with the process of conducting a systematic review and to present them with the lay summary of the findings. Then we asked them an open-ended question “Do the results make sense to you?” To respect time and schedule of our patient advisers, we provided each of them the opportunity to answer in the manner they felt most comfortable with (e.g., in-person, phone, e-mail). Throughout each of our correspondence, patient-advisers were provided with the opportunity to seek clarification or ask questions about the results and the study itself.

## Results

### Search Results

The search returned 6,869 articles. After removing duplicates, titles and abstracts of 5,416 articles were screened. Only 23 articles were eligible for full-text review. Implementation and use of PROMs outside clinical care, focus on acute diseases, and descriptive type of article were the primary reasons for excluding most of the studies. During full-text review, five articles from four studies met the inclusion criteria, where two articles were written based on the same study but reported different types of outcomes in two separate articles. Information provided in conference abstracts was deemed inadequate, so they were excluded. Hand-search of reference list of included studies and Web of Science Database identified two more articles. Thus, a total of seven articles from six studies were included for final analysis. PRISMA Flow Diagram (Figure 1) outlines the number of records identified, included, and excluded and selection of articles through the different phases of this systematic review.

### Characteristics of the Included Studies

All the identified studies were published between 2008 and 2017 and the duration of studies ranged from 5 to 45 months. Five of the six studies were conducted in European countries (Netherlands, Germany, and Spain), while one study was conducted in the United States of America. Diseases of interest varied for each study with a range of chronic diseases including cancers, rheumatic and juvenile idiopathic arthritis, asthma, and diabetes. Three of the included studies were randomized control trials, two employed a sequential cohort model, and one was a cohort study followed by a mixed methods component. Healthcare providers receiving the results of PROMs were mostly pediatric subspecialists. Both generic and disease-specific PROMs were used. The PROMs evaluated in these studies are listed in Table 1.

While all the studies utilized electronic platforms to administer PROMs, the frequency of administration varied from once a week to quarterly. Finally, the evaluated outcomes for each study varied substantially. Identification of HRQOL domains and communication about those domains were the most commonly evaluated outcomes (Table 2). Other reported outcomes were HRQOL scores, satisfaction among parents and healthcare providers, duration of consultations, and referral rates. Included studies did not provide information on the effectiveness of integrating generic vs. disease-specific PROMs; therefore, we did not conduct subgroup analysis based on the type of PROM.

### Impact on Outcomes

A narrative analysis of the impact of PROMs on various outcomes is presented below and is discussed in more detail in the subsequent section.

#### HRQOL

Four of the six included studies evaluated the impact of using PROMs on HRQOL score in routine pediatric clinical care and reported an increase in the overall score of HRQOL among the intervention group. The positive change in HRQOL reported by Engelen et al. (31) and Murillo et al. (36) was statistically significant. Murillo et al. (36) reported that periodic evaluation of HRQOL as part of the routine visit of diabetes patients improved HRQOL, especially in the domains of psychological well-being and school environment. de Wit et al. (30) reported that for adolescent diabetes patients with lower A1C levels, scores of the HRQOL intervention group improved, whereas HRQOL score remained stable in the control group.

#### Identification and Communication Around HRQOL Domains

Five of the six included studies evaluated and reported a positive impact of using PROMs on identification and communication around HRQOL domains among the intervention group. While all five studies reported some improvement in identification and communication around issues in HRQOL domains in the intervention group, only Engelen et al. (31) reported a statistically significant improvement in communication around HRQOL domains. de Wit et al. (30) showed a statistically significant effect in the intervention group (especially in behavior and self-esteem subscales); however, adolescents with relatively high A1C values at baseline did not show any improvements (nor worsening) of psychosocial outcomes over time.

#### Satisfaction With Using PROMs in Routine Clinical Care

Five of the six studies evaluated the impact on quality of care. Engelen et al. (31) and Haverman et al. (40) found a trend toward higher scores for satisfaction with care among the intervention group, and their multilevel analysis demonstrated no difference in satisfaction between the intervention and control groups. Haverman et al. (40) found no difference in satisfaction among patients or parents between the intervention and control groups but reported higher satisfaction among healthcare providers in the intervention group (pediatric rheumatologists). Although de Wit et al. (30) reported a statistically significant improvement in the satisfaction in intervention group, their linear regression analysis showed that increased satisfaction with care was independent of changes in HRQOL. Wolfe et al. (34) and Barthel et al. (35) evaluated satisfaction with using PROMs as an intervention in routine clinical care. Wolfe et al. (34) reported that half of pediatric patients found PROM reports easy to understand, but only a quarter of total patients thought the reports helped them *quite a bit* or *very much* with talking to their doctors. While almost two-thirds of parents found PROM reports easy to understand, only half of the parents thought reports helped them *quite a bit/very* much with talking to their child's doctors. However, ~2/3rd of healthcare providers agreed that PROM reports (through PediQUEST system) were at least somewhat helpful, and more so than e-mail alerts (34). Barthel et al. (35) reported that children and adolescents perceived their electronic platform (Kids-CAT) to be a highly feasible and user-friendly tool for providing HRQOL scores. Similarly, healthcare providers reported that integrating PROMs in their routine clinical care had a positive influence on patient–physician communication.

#### Duration of Consultation

Three of the six included studies reported the impact of using PROMs on the average duration of consultation. This outcome showed mixed results. Engelen et al. (31, 32) showed a reduction in the duration of consultation, but Wolfe et al. (34) found that using PROMs did not increase total consultation time, and Barthel et al. (35) reported a mix of conflicting results and overall low potential to save time.

#### Referral Rate

Three of the six studies evaluated and reported the impact of using PROMs on the referral rate. While Haverman et al. (40) and Wolfe et al. (34) reported an increase in the referral rates in the intervention group, Engelen et al. (31) showed no difference in referral rates between the intervention and control groups.

#### Other Outcomes

Only Murillo et al. (36) and de Wit et al. (30) reported the impact of using PROMs on clinical outcomes for patients with type-1 diabetes. Both these studies did not find any difference in HbA1c levels between the intervention and control groups over time (30, 36). None of the studies evaluated or reported the impact of using PROMs on healthcare utilization.

### Risk of Bias Assessment

In accordance with Downs and Black's checklist, the quality scores are provided in Table 1. The scores from all studies ranged between 15 and 22. Overall, the methodological quality of the included studies indicated a moderate risk of bias and was determined to be of moderate quality.

### Results From Patient-Advisers' Perspective

During our consultations, our patient-advisers agreed with most of the findings of this systematic review, except the referral rate. According to them, the referral rates should have decreased because integrating using PROMs should support clinicians providing a holistic care including patient's clinical and psychosocial needs, which could potentially avoid unnecessary referrals to other specialists.

## Discussion

Our systematic review suggests that integrating PROMs in routine pediatric clinical care positively impacts HRQOL while enhancing identification and communication around HRQOL domains. Integrating PROMs in routine pediatric clinical care is also positively associated with satisfaction. Duration of consultation and reference rates showed a mix of conflicting results. Two studies [de Wit et al. (30) and Murillo et al. (36)] reporting the impact of integrating PROMs in routine pediatric clinical care on clinical outcomes did not show any difference between intervention and control groups over time.

All the studies except Wolfe et al. (34) were conducted only within outpatient settings. This systematic review also revealed that integration of PROMs in routine pediatric clinical care is more prevalent in European countries; in fact, all the studies except Wolfe et al. (34) were conducted among European countries, mostly in the Netherlands where three of the six studies were conducted. These two findings highlight a major gap in our knowledge of the impact of integrating PROMs within outpatient pediatric clinical care and in jurisdictions outside Europe.

Our systematic review shows that integrating PROMs in routine pediatric clinical care could help healthcare providers to identify issues in various HRQOL domains, which enhances communication about associated issues, with improvements in HRQOL. Integration of PROMs in routine clinical care helps to deliver patient and family-centered care by focusing on the patient's health goals and guiding therapeutic decisions, which produces better health outcomes and enhances patient and family satisfaction (27, 41).

One of the outcomes of interest for this systematic review was hospitalization, but none of the studies evaluated the impact of integrating PROMs in routine pediatric clinical care on hospitalization as an outcome. Hospitalization is a crucial indicator for utilization of health services. In fact, hospitalization associated with chronic diseases can have a detrimental impact on the social and economic status of the family, which could have long-term consequences that endure into adulthood (42, 43). Thus, future studies should consider evaluating the impact of integrating PROMs in routine pediatric clinical care on healthcare utilization.

One study [Engelen et al. (31)] showed lower average duration of consultation. Our patient-advisers also expressed the views that integrating PROMs would help healthcare providers ask more targeted questions, which could reduce the consultation time. However, Barthel et al. (35) reported that healthcare providers in one of the focus groups felt that integrating PROMs has a low potential to save time (35). Wolfe et al. (34) also reported mixed results. Similarly, the referral rate was either constant or increased in the intervention group which was contested by our patient-advisers who believed integrating PROMs in routine clinical care should decrease the number of referrals. While this contradiction offers a unique observation, it also provides an encouraging opportunity to engage patient-advisers in evidence synthesis. These conflicting results for referral rate and consultation time should be further investigated in future studies.

PROMs have traditionally been administered in paper format, but the use of electronic PROM collection systems is on the rise (44). The International Society for Pharmacoeconomics and Outcomes Research has formulated specific guidelines to ensure there is equivalence between paper and electronic versions of PROMs (23). Our systematic review suggests a similar trend of using electronic PROMs. Routine collection of “patient-centered data” provides learning health care systems with the opportunity to link these data to other databases, including electronic health records and administrative data, which can then be used to assess the performance of a healthcare system and to identify areas for improvements or used for comparative effectiveness research (45, 46). In clinical settings, these data could be utilized to provide evidence-based personalized care (33, 47, 48).

Recently, Miller et al. (49) highlighted the time-constrained, resource-limited nature of contemporary healthcare settings and the fragmentation of provider–provider IT systems as potential barriers to operationalize PROMs in routine clinical care. However, none of the studies included in our systematic review explicitly report any barriers to the integration PROMs in routine pediatric clinical care.

Comparing the quality of studies with different study designs was challenging. All the studies included in this systematic review are of moderate quality which could also be due to that fact that Downs and Black's checklist is influenced by the study design as it gives a higher score to randomized controlled trials while other study designs receive lower scores.

### Strengths and Limitations

The strength of this systematic review lies in the rigorous methodology applied throughout its conduct. Soliciting the perspectives of patient-advisers by presenting them with the results of this systematic review is a novel approach, and we believe that this is a strength of this review. This additional step helped us to understand the appropriateness, sensibility, and relevance of the results of this systematic review as they would appear to the patients and their family caregivers. While we realize that our approach to solicit feedback from our patient-advisers might be considered a weakness, we do feel, however, that this is an advancement toward patient-oriented research in pediatric settings, albeit not a perfect one. As a systematic review, we did not include any studies from the gray literature, and so potential information in other reporting contexts regarding PROMs implementation could have been missed.

## Conclusion

Our systematic review confirms findings from studies conducted in adult populations. Integrating PROMs in routine pediatric clinical care could have a positive impact on HRQOL. However, considering the quality of the studies included in this review, more randomized controlled trials are warranted. Trends in the results around satisfaction show that patients and their caregivers and healthcare providers are generally more satisfied with using PROMs. Integration of PROMs increased identification and discussion around HRQOL, especially in psychosocial and emotional domains. We would recommend future systematic reviewers to devise more rigorous approaches to engage patient-advisers throughout the conduct of the review. Our systematic review highlights a significant gap in the literature focusing on pediatric populations. Future studies should evaluate the impact of integrating PROMs in routine pediatric clinical care on key outcomes including healthcare utilization, quality of care, clinical outcomes, and average duration of consultation.

## Data Availability Statement

The original contributions presented in the study are included in the article/Supplementary Material, further inquiries can be directed to the corresponding author/s.

## Table of Contents Summary

In this systematic review, we evaluate the impact of integrating patient-reported outcome measures in routine clinical care of pediatric patients with chronic diseases.

## Author Contributions

MS conceptualized and designed the study, coordinated, and supervised data collection, and reviewed and revised the manuscript. SB conceptualized and designed the study, designed the data collection instruments, collected data, carried out the initial analyses, drafted the initial manuscript, and revised the manuscript. AC, BM, and LT designed the data collection instruments, collected data, and reviewed and revised the manuscript. LH critically reviewed the manuscript for important intellectual content. All authors approved the final manuscript as submitted and agree to be accountable for all aspects of the work.

## Conflict of Interest

The authors declare that the research was conducted in the absence of any commercial or financial relationships that could be construed as a potential conflict of interest.

## Abbreviations

PCC: patient-centered care
HRQOL: health-related quality of life
PRO: patient-reported outcome
PROM: patient-reported outcome measure
PRISMA: preferred reporting items for systematic review and meta-analysis
COSMIN: COnsensus-based Standards for the selection of health status Measurement Instruments
ISPOR: International Society for Pharmacoeconomics and Outcomes Research
PedsQL4.0: Pediatric Quality of Life Inventory 4.0.
