# Supplementary material for: Patient-Reported Outcome Measures in Routine Pediatric Clinical Care: A Systematic Review
Source: Front Pediatr. 2020 Jul 28;8:364. doi: 10.3389/fped.2020.00364 (PMC7399166; doi:10.3389/fped.2020.00364)
Supplement: Supplementary file 1 [file Table_1.DOCX]

**Appendix 1: Search strategy**

**Population terms**

1. adolescen*.tw,kf.

2. child*.tw,kf.

3. (p?ediatric adj care).tw,kf.

4. Child Health Services/

5. Child/

6. Adolescent/

7. Child, Hospitalized/

8. Adolescent, Hospitalized/

9. or/1-8

**Intervention terms**

10. patient outcome assessment*.tw,kf.

11. patient reported outcome measure*.tw,kf.

12. patient reported treatment outcome*.tw,kf.

13. (PROs or PROMs or PROMIS).tw,kf.

14. self-report* measure*.tw,kf.

15. self-report* outcome*.tw,kf.

16. Patient Reported Outcome Measures/

17. patient reported outcome*.tw,kf.

18. or/10-17

**Outcomes terms**

19. (emergency adj (admission* or attendance or attender* or readmission* or re-admission* or visit*)).tw,kf.

20. (hospital adj (admission* or readmission* or re-admission* or visit*)).tw,kf.

21. hospital length of stay.tw,kf.

22. Emergency Service, Hospital/ut, td, sn, ec [Utilization, Trends, Statistics & Numerical Data, Economics]

23. Hospitalization/

24. hospitali*.tw,kf.

25. length of stay/

26. Patient Admission/

27. Patient Readmission/

28. (quality adj2 (care or healthcare)).tw,kf.

29. (quality adj2 health adj2 care).tw,kf.

30. Quality Indicators, Health Care/

31. Quality of Health Care/

32. quality of life.tw,kf.

33. HRQOL.tw,kf.

34. Quality of Life/

35. *nurse-patient relations/ or *physician-patient relations/

36. or/19-35

**All Combined**

37. 9 and 18 and 36

38. limit 37 to (yr="2000 -Current" and english)
